# *endo*,*endo*-Tetra­cyclo­[6.2.1.1^3,6^.0^2,7^]dodeca-9-en-*anti*-11-yl 4-bromo­benzoate

**DOI:** 10.1107/S1600536812051902

**Published:** 2013-01-09

**Authors:** Barry A. Lloyd, Atta M. Arif, Robert J. Coots

**Affiliations:** a Chemistry Department, Weber State University, Ogden, Utah 84408-2503, USA; b Chemistry Department, University of Utah, Salt Lake City, Utah 84112, USA; c Colonial Chemical, Inc., 225 Colonial Drive, South Pittsburg, Tennessee 37380, USA

## Abstract

The title compound 1-OPBB, C_19_H_19_BrO_2_, contains a dechlorinated and hydrogenated isodrin backbone with an *anti*-4-bromo­benzoate substituent at one of the methano bridges. The dihedral angle between the CO_2_ ester plane and the benzene ring plane is 8.5 (2)°. In the crystal, the ester groups stack over benzene rings: the mol­ecules pack as conformational enanti­omers, with nearest parallel benzene ring planes separated by a perpendicular distance of 3.339 (1) Å. The nearest benzene-ring centroids are 5.266 (1) Å apart. Possible structural correlation with enhanced solvolytic reactivity is investigated.

## Related literature

For related norbornyl and norbornenyl 4-bromo­benzoate structures, see: Lloyd & Arif (2012*a* ▶,*b* ▶). For a structure containing the same tetra­cyclic framework, see: Lloyd *et al.* (1995 ▶). For the isomeric *endo,exo*-structure, see: Lloyd *et al.* (1994 ▶). For solvolysis rate information, see: Coots (1983 ▶); Chow & Jiang (2000 ▶). For mol­ecular orbital results, see: Furusaki & Matsumoto (1978 ▶); Chow (1998 ▶, 1999 ▶). For synthetic procedures, see: Chow (1996 ▶); Melder & Prinzbach (1991 ▶); Coots (1983 ▶).
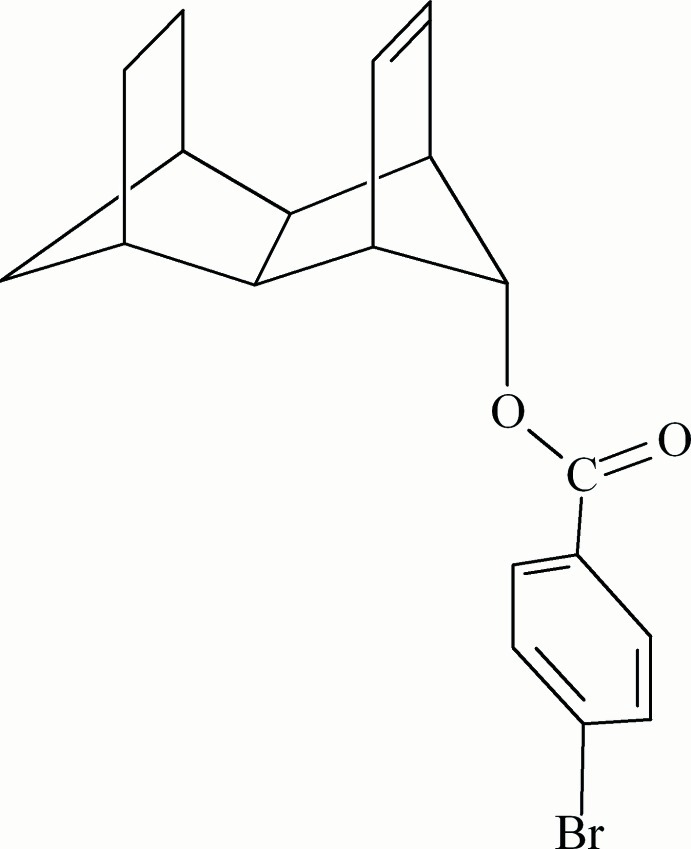


## Experimental

### 

#### Crystal data

C_19_H_19_BrO_2_
*M* *_r_* = 359.25
Monoclinic, 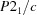

*a* = 13.2569 (2) Å
*b* = 10.5045 (2) Å
*c* = 12.2039 (2) Å
β = 116.0122 (9)°
*V* = 1527.32 (4) Å^3^
*Z* = 4
Mo *K*α radiation
μ = 2.70 mm^−1^
*T* = 150 K
0.23 × 0.20 × 0.13 mm

#### Data collection

Nonius KappaCCD diffractometer
Absorption correction: multi-scan (*DENZO-SMN*; Otwinowski & Minor, 1997 ▶) *T*_min_ = 0.576, *T*_max_ = 0.721
6702 measured reflections
3500 independent reflections
2700 reflections with *I* > 2σ(*I*)
*R*_int_ = 0.021

#### Refinement

*R*[*F* ^2^ > 2σ(*F* ^2^)] = 0.028
*wR*(*F* ^2^) = 0.063
*S* = 1.03
3500 reflections
276 parameters
All H-atom parameters refined
Δρ_max_ = 0.40 e Å^−3^
Δρ_min_ = −0.46 e Å^−3^

### 

Data collection: *COLLECT* (Nonius, 1998 ▶); cell refinement: *DENZO-SMN* (Otwinowski & Minor, 1997 ▶); data reduction: *DENZO-SMN*; program(s) used to solve structure: *SIR97* (Altomare *et al.*, 1999 ▶); program(s) used to refine structure: *SHELXL97* (Sheldrick, 2008 ▶); molecular graphics: *WinGX* (Farrugia, 2012 ▶), *ORTEP-3* (Farrugia, 2012 ▶) and *PLATON* (Spek, 2009 ▶); software used to prepare material for publication: *Mercury* (Macrae *et al.*, 2008 ▶) and *publCIF* (Westrip, 2010 ▶).

## Supplementary Material

Crystal structure: contains datablock(s) I, global. DOI: 10.1107/S1600536812051902/hb7005sup1.cif

Structure factors: contains datablock(s) I. DOI: 10.1107/S1600536812051902/hb7005Isup2.hkl

Supplementary material file. DOI: 10.1107/S1600536812051902/hb7005Isup3.mol

Supplementary material file. DOI: 10.1107/S1600536812051902/hb7005Isup4.cml

Additional supplementary materials:  crystallographic information; 3D view; checkCIF report

## Figures and Tables

**Table 1:** Possible structure/reactivity relationships (°, Å)

	1-OPBB	2-OPBB	3-OPBB	4-OPBB	5-OPBB
Solvolysis rate*^*a*^*	210	480	28	1.0	10^−11^
1:2 inter­planar angle*^*b*^*	121.9 (2)	119.8 (6)	122.9 (3)	124.5 (1)	121.2 (1)
3:4 inter­planar angle	132.0 (1)	132.4 (4)	128.1 (2)		
C11—O2 bond length*^*b*^*	1.450 (2)	1.460 (7)	1.437 (3)	1.445 (2)	1.447 (2)

Notes: (*a*) Rates determined in 80% dioxane-*d*_8_/20% D_2_O at 383 K, from NMR peak integrations; (*b*) C1/C7/C4 is plane 1 and C1/C2/C3/C4 is plane 2 for 4-OPBB and 5-OPBB; bond length is C7—O2 for 4-OPBB and 5-OPBB.

**Fig. 1.**
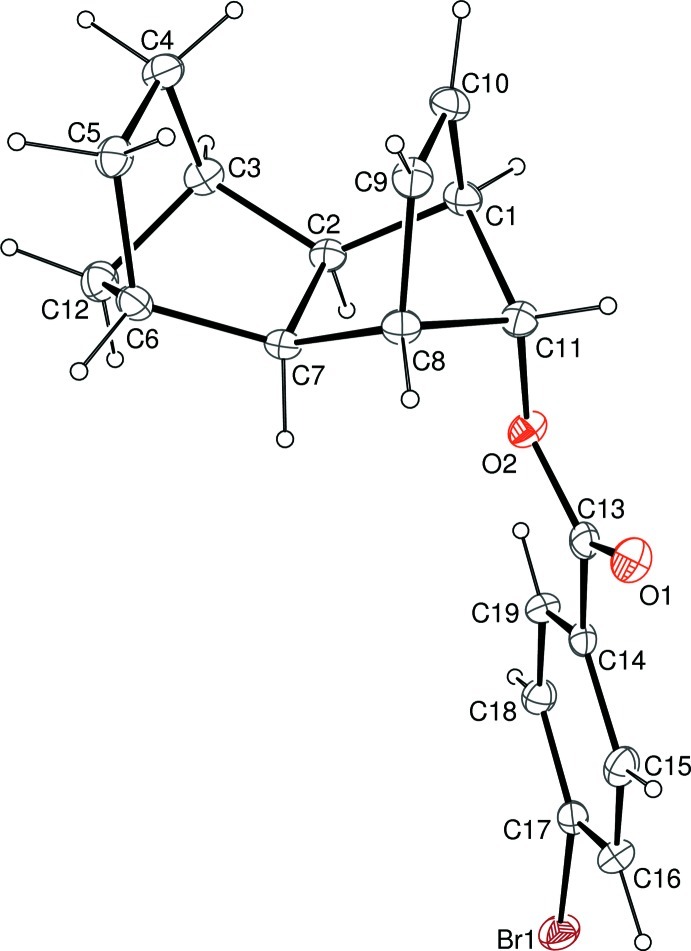
ORTEP-3 drawing of the title compound showing 50% displacement ellipsoids.

**Fig. 2.**
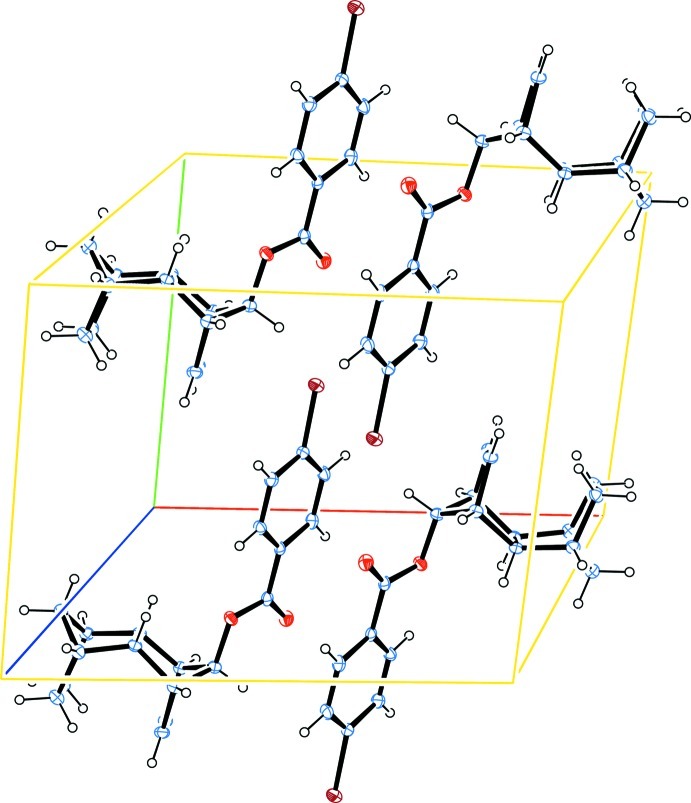
Cell packing diagram for the title compound.

**Fig. 3.**
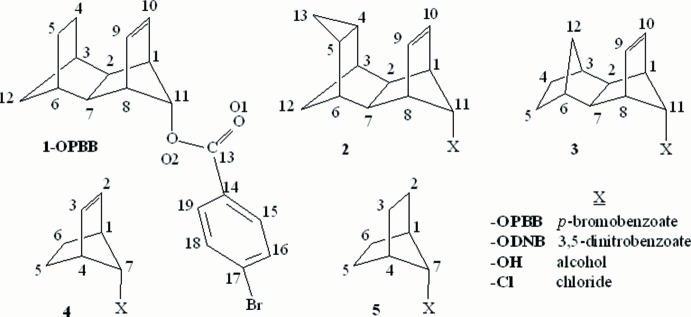
Compound 1- to 5-OPBB structures.

**Fig. 4.**
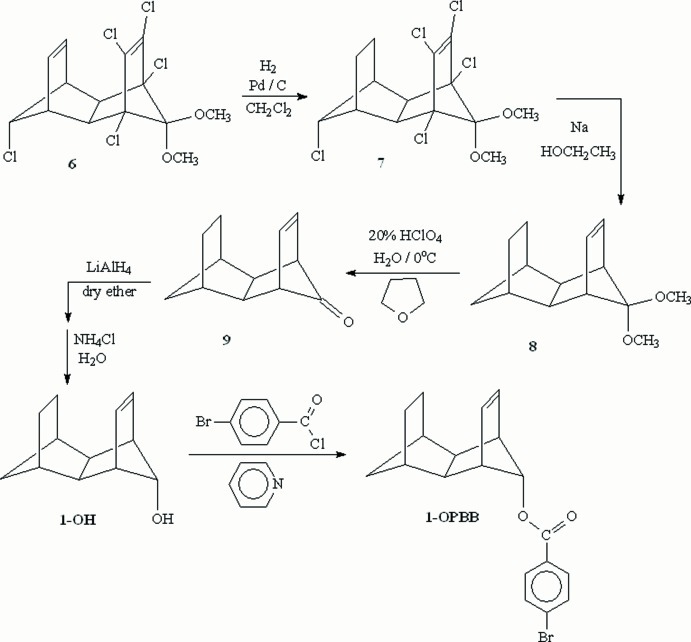
Synthesis scheme for 1-OPBB.

## supplementary crystallographic information

### Comment

An *ORTEP*-3 drawing (Farrugia, 2012) and a cell packing diagram of the title compound, 1-OPBB, are shown in Figs. 1 and 2, respectively. A Cambridge Structural Database search found only one other structure (2-OPBB) containing this tetracyclic monoene structure (Lloyd *et al.*, 1995).

3,5-Dinitrobenzoate esters 1-, 2- and 3-ODNB (Fig. 3) solvolyze faster (Table 1) in 80% dioxane-*d*_8_/20% D_2_O at 383 K than 4-ODNB (Coots, 1983). The 1-ODNB rate increase was explained by long range σ-orbital through-space and through-bond mixing with the homoconjugated π-system, that stabilizes the intermediate carbocation (Chow & Jiang, 2000, Chow, 1999, Chow, 1998, Furusaki & Matsumoto, 1978). The 1-OPBB X-ray crystal structure provides experimental verification for some of the calculated results.

No nonhydrogen atom intermolecular contacts exist in 1-OPBB shorter than van der Waals radii sums, the closest being C13^i^···C14^ii^ at 3.430 (3) Å [symmetry code: (ii) 1 - *x*, -*y*, 1 - *z*]. The closest tetracyclic hydrogen intermolecular contact is H3^i^···H12B^iii^ 2.34 (3) Å [symmetry code (iii) -*x*, -*y*, 1 - *z*]. Least squares planes are defined as C1—C11—C8 (plane 1), C1—C10—C9—C8 (plane 2), C1—C2—C7—C8 (plane 3), C2—C3—C6—C7 (plane 4), C3—C4—C5—C6 (plane 5), C3—C12—C6 (plane 6), H9—C9—C10—H10 (plane 7), C14—C15—C16—C17—C18—C19 (plane 8) and O1—C13—O2 (plane 9). Interplanar angles are: 1:2 121.9 (2)°, 1:3 119.9 (1)°, 2:3 118.2 (1)°, 3:4 132.0 (1)°, 4:5 119.1 (1)°, 4:6 119.6 (1)°, 5:6 121.4 (1)°, and 8:9 8.5 (2)*^o^*. The largest carbon atom deviation from planarity is 0.003 (1) Å in plane 5. The greatest difference between symmetry-related bond lengths is 0.011 (4) Å (for C1—C11 *versus* C8—C11, and between symmetry-related bond angles is 0.78 (20)° (between C2—C7—C6 and C3—C2—C7). Bonds C1—C2, C2—C7, C4—C5 and C7—C8 are somewhat longer than usual, similar to analogous bonds in 2- (Lloyd *et al.*, 1995), isomeric 3- (Lloyd, *et al.*, 1994), 4-, and 5-OPBB (Lloyd & Arif, 2012*a*,*b*). Less alkenic C pyramidalization is apparent in 1-OPBB (2:7 angle 2 (1)°) than in 3-OPBB (comparable 2:4 angle (6 (1)°).

The 1:2 interplanar angle (Table 1) is a logical structure/solvolysis reactivity indicator for these compounds. A smaller angle should portend faster solvolysis as the homoconjugated π-bond provides anchimeric assistance. Solvolytic reactivities are inverse to 1:2 angles for 1-, 2-, 3-, and 4-OPBB, but differences are small relative to the calculated ~30° substrate to transition state 1:2 angle bending (Chow, 1999 and Chow, 1998), and other structural features are certainly involved. The 1-OPBB 3:4 angle is near that of *endo,endo*2-OPBB, and larger than in 3-OPBB, consistent with more interbridge C4—C5···C9═C10 steric repulsion in *endo,endo* than in *endo,exo* structures. The 1-OPBB 4:6 angle is 1.9 (5)° larger, and the 5:6 angle is 5.2 (8)° smaller than in 2-OPBB, which probably reflects the close C12···C13 contact (2.70 (1) Å) in the latter. A longer C11—O2 (structures 1-, 2-, 3-OPBB) or C7—O2 (structures 4- and 5-OPBB) bond should also imply faster solvolysis, but they do not fit the expected pattern for 3- and 5-OPBB.

Short intramolecular van der Waals contacts demonstrate C4—C5···C9═C10 and C9═C10···C11 steric interactions: C4···C10 3.014 (4), C5···C9 2.993 (3), H5B···C9 2.39 (3), H4B···C10 2.45 (3), C9···C11 2.284 (3) and C10···C11 2.279 (3) Å. Theoretical values for 1-Cl (Chow, 1999 and Chow, 1998) agree closely: C4···C10 (and C5···C9) 2.94 (semiempirical AM1) or 3.00 Å (*ab initio* HF/3–21 G), C9···C11 (and C10···C11) 2.33 (AM1) or 2.30 Å (HF/3–21 G), and interplanar 1:2 angle 122° (HF/3–21 G).

### Experimental

Compound 1-OPBB was synthesized *via* the steps shown in Fig. 4 and described below. Products were verified by 90 MHz ^1^H NMR spectroscopy in CDCl_3_ solvent. The most recent methods for synthesizing precursor compound 6 are found in Chow (1996). See also Melder & Prinzbach (1991) for substrate syntheses.

Into 70 ml of CH_2_Cl_2_ were dissolved 2.0 g of 6 and 0.1 g of 10% Pd/C was added. The mixture was stirred under H_2_ (~9 × 10 ^4^ Pa) at 298 K for 12 h. The mixture was vacuum filtered to remove catalyst, and CH_2_Cl_2_ was removed under vacuum yielding 2.0 g of white powder 7: mp 400.5 - 401.5 K. ^1^H NMR: δ 1.58 (4*H*, m), 2.52 (2*H*, m), 3.50 (2*H*, m), 3.68 (3*H*, s), 3.75 (3*H*, s), 4.22 (1*H*, m).

Into 100 ml of absolute ethanol were dissolved 6.7 g of 7. Over a 2 h period, 19.6 g of Na (washed twice in absolute ethanol) were added as small (~0.3 g) pieces under a dry, N_2_ atmosphere while refluxing and mechanically stirring. After 6 h the mixture was cooled to 298 K and 200 g of crushed ice were slowly and cautiously added while stirring. The mixture was extracted with 3 × 100 ml of ether, and combined ether extracts were washed with water, saturated brine, and dried over MgSO_4_. Solvent removal under vacuum yielded 3.2 g (85%) of pale yellow oil 8.^1^H NMR: δ 1.22 (4*H*, bs), 1.28 (1*H*, m), 1.46 (1*H*, d, *J* \sim 9 Hz), 2.09 (2*H*, bs), 2.41 (2*H*, m), 2.72 (2*H*, m), 3.13 (3*H*, s), 3.18 (3*H*, s), 6.03 (2*H*, m).

Into 50 ml of tetrahydrofuran were dissolved 3.0 g of 8. The solution was cooled to 273 K and poured into 35 ml of 20% aqueous HClO_4_ in an ice bath. The mixture warmed to 298 K overnight, was then poured into 100 ml of water, and extracted with 3 × 50 ml of ether. Combined ether extracts were washed with water, saturated NaHCO_3_, saturated brine, and dried over MgSO_4_. Ether was evaporated under vacuum yielding 2.2 g of colorless oil 9 that crystallized upon standing: mp 330.0–331.5 K. ^1^H NMR: δ 1.18 (4*H*, bs), 1.29 (2*H*, m), 2.23 (2*H*, m), 2.32 (2*H*, m), 2.93 (2*H*, m), 6.34 (2*H*, m).

Into 100 ml of absolute ether were placed 0.126 g of LiAlH_4_ under a dry N_2_ atmosphere. After stirring for 1 h at 298 K, the mixture was cooled to 195 K and a 2.1 g solution of 9 in 20 ml of absolute ether was slowly added over 30 min. The mixture was stirred for 1 h at 195 K, and allowed to warm up overnight. Excess LiAlH_4_ was then neutralized by slowly adding 0.9 ml of saturated aqueous NH_4_Cl and stirring 30 min. About 1 g of MgSO_4_ was added and the mixture stirred 30 min more. Vacuum filtration removed solids. Ether was evaporated under vacuum, yielding 1.5 g (70%) of white 1-OH crystals which were further purified by preparative gas chromatography (1.5 m × 0.0063 m stainless steel column, 20% DEGS on 60/80 Chromosorb W AW, injector 483 K, column 438 K, detector 483 K, He carrier 75 ml/min, 1-OH retention time 5.85 min): mp 406.5–407.5 K. ^1^H NMR: δ 1.28 (4*H*, bs), 1.40 (1*H*, d, *J*~9 Hz), 1.66 (1*H*, d, *J*~9 Hz), 1.92 (1*H*, bs), 2.13 (2*H*, m), 2.44 (2*H*, m), 2.54 (2*H*, m), 3.72 (1*H*, bs), 5.98 (2*H*, m). ^13^C NMR (CDCl_3_, 20 MHz): δ 25.9, 39.4, 46.0, 47.46, 47.50, 90.7, 129.8.

Into 5 ml of freshly distilled dry pyridine (from CaH_2_) were dissolved 0.086 g of pure 1-OH, and 0.14 g of freshly recrystallized (from hexanes) 4-bromobenzoyl chloride with stirring under a dry N_2_ atmosphere. The mixture was warmed briefly until reagents dissolved, and stirred overnight at 298 K. The mixture was poured into 100 ml of cold water, and extracted with 2 × 50 ml of ether. Combined ether extracts were washed with water, twice with 10% HCl, twice with NaHCO_3_, and with saturated brine. The ether solution was dried over MgSO_4_, filtered, and ether was evaporated under vacuum, yielding crude 1-OPBB. Recrystallization from a 1:4 CHCl_3_ / hexane mixture yielded 0.15 g (86%) of white 1-OPBB crystals. These were dissolved in ~5 ml of CH_2_Cl_2_ and passed down a 0.05 m × 0.005 m silica gel column, eluting with distilled CH_2_Cl_2_, and solvent was evaporated. The residual white crystals were sublimed (353 K, 1.3 Pa) yielding pure white 1-OPBB crystals: mp 409.5–410.5 K. ^1^H NMR: δ 1.30 (4*H*, bs), 1.43 (1*H*, d, *J*~9 Hz), 1.68 (1*H*, d, *J*~9 Hz), 2.18 (2*H*, m), 2.65 (2*H*, m), 2.83 (2*H*, m), 4.76 (1*H*, bs), 6.21 (2*H*, m), 7.76 (2*H*, d, *J*~9 Hz), 8.05 (2*H*, d, *J*~9 Hz).

About 0.1 g of sublimed 1-OPBB was dissolved in 15 ml of absolute ethanol by warming on a steam bath for 5 min. This solution was placed in a crystallizing dish and covered with plastic wrap. Three small holes were made in the plastic wrap with a hot wire and ethanol slowly evaporated at 298 K. About ten crystals were eventually removed from the evaporating dish, and one of these was selected for the X-ray structure analysis.

### Refinement

A colorless plate shaped crystal 0.23 × 0.20 × 0.13 mm in size was mounted on a quartz fiber with epoxy resin, and transferred to a Nonius KappaCCD diffractometer equipped with Mo Kα radiation (λ = 0.71073 Å). Ten frames of data were collected at 150 (1) K with an oscillation range of 1°/frame and an exposure time of 20 sec/frame (Nonius, 1998). Indexing and unit cell refinement based on all observed reflections from those ten frames indicated a monoclinic *P* lattice. A total of 6702 reflections (Θ_max_ = 27.49°) were indexed, integrated and corrected for Lorentz, polarization and absorption effects using *DENZO– SMN* and *SCALEPAC* (Otwinowski & Minor, 1997). Post refinement of the unit cell gave *a* = 13.2569 (2) Å, *b* = 10.5045 (2) Å, *c* = 12.2039 (2) Å, β = 116.0122 (9)°, and V = 1527.32 (4) Å^3^. Axial photographs and systematic absences were consistent with the compound having crystallized in the monoclinic space group *P*2_1_/c.

The structure was solved by a combination of direct and heavy atom methods using *SIR97* (Altomare *et al.*, 1999). All of the non-hydrogen atoms were refined with anisotropic displacement coefficients. Hydrogen atoms were located and refined isotropically using *SHELXL97* (Sheldrick, 2008). The weighting scheme employed was *w* = 1/[σ^2^(*F*_o_^2^) + (0.0223*P*)^2^ + 0.8628*P*] where *P* = (*F*_o_^2^ + 2*F*_c_^2^) /3. The refinement converged to *R*1 = 0.0276, *wR*2 = 0.0577, and *S* = 1.029 for 2700 reflections with *I* > 2σ(*I*), and *R*1 = 0.0452, *wR*2 = 0.0632 and *S* = 1.029 for 3500 unique reflections and 276 parameters, where *R*1 = Σ (|| *F*_o_ | – |*F*_c_ ||) / Σ |*F*_o_|, *wR*2 = [Σ(*w*(*F*_o_^2^ – *F*_c_^2^)2) / Σ(*F*_o_^2^)^2^]^1/2^, and *S* = Goodness-of-fit on *F*^2^ = [Σ (*w*(*F*_o_^2^ – *F*_c_^2^)^2^ / (*n*-*p*)]^1/2^; *n* is the number of reflections and *p* is the number of parameters refined. The maximum Δ/σ in the final cycle of the least-squares was 0.001, and the residual peaks on the final difference-Fourier map ranged from -0.457 to 0.397 e/Å^3^. Scattering factors were taken from the International Tables for Crystallography, Volume C, Chapters 4 pp 206–222 and 6 pp 476–516.

### Crystal data

**Table:**

C_19_H_19_BrO_2_	*F*(000) = 736
*M**_r_* = 359.25	*D*_x_ = 1.562 Mg m^−^^3^
Monoclinic, *P*2_1_/*c*	Melting point = 409.5–410.5 K
Hall symbol: -P 2ybc	Mo *K*α radiation, λ = 0.71073 Å
*a* = 13.2569 (2) Å	Cell parameters from 3686 reflections
*b* = 10.5045 (2) Å	θ = 1.0–27.5°
*c* = 12.2039 (2) Å	µ = 2.70 mm^−^^1^
β = 116.0122 (9)°	*T* = 150 K
*V* = 1527.32 (4) Å^3^	Plate, colourless
*Z* = 4	0.23 × 0.20 × 0.13 mm

### Data collection

**Table:**

Nonius KappaCCD diffractometer	3500 independent reflections
Radiation source: fine-focus sealed tube	2700 reflections with *I* > 2σ(*I*)
Graphite monochromator	*R*_int_ = 0.021
Phi and ω scan	θ_max_ = 27.5°, θ_min_ = 2.6°
Absorption correction: multi-scan (*DENZO-SMN*; Otwinowski & Minor, 1997)	*h* = −17→17
*T*_min_ = 0.576, *T*_max_ = 0.721	*k* = −13→13
6702 measured reflections	*l* = −15→15

### Refinement

**Table:**

Refinement on *F*^2^	Secondary atom site location: difference Fourier map
Least-squares matrix: full	Hydrogen site location: inferred from neighbouring sites
*R*[*F*^2^ > 2σ(*F*^2^)] = 0.028	All H-atom parameters refined
*wR*(*F*^2^) = 0.063	*w* = 1/[σ^2^(*F*_o_^2^) + (0.0223*P*)^2^ + 0.8628*P*] where *P* = (*F*_o_^2^ + 2*F*_c_^2^)/3
*S* = 1.03	(Δ/σ)_max_ = 0.001
3500 reflections	Δρ_max_ = 0.40 e Å^−^^3^
276 parameters	Δρ_min_ = −0.46 e Å^−^^3^
0 restraints	Extinction correction: *SHELXL97* (Sheldrick, 2008), Fc^*^=kFc[1+0.001xFc^2^λ^3^/sin(2θ)]^-1/4^
Primary atom site location: structure-invariant direct methods	Extinction coefficient: 0.0021 (3)

### Special details

**Table:**

Experimental. The program *DENZO-SMN* (Otwinowski & Minor, 1997) uses a scaling algorithm which effectively corrects for absorption effects. High redundancy data were used in the scaling program hence the 'multi-scan' code word was used. No transmission coefficients are available from the program (only scale factors for each frame). The scale factors in the experimental table are calculated from the 'size' command in the *SHELXL-97* input file.
Geometry. All e.s.d.'s (except the e.s.d. in the dihedral angle between two l.s. planes) are estimated using the full covariance matrix. The cell e.s.d.'s are taken into account individually in the estimation of e.s.d.'s in distances, angles and torsion angles; correlations between e.s.d.'s in cell parameters are only used when they are defined by crystal symmetry. An approximate (isotropic) treatment of cell e.s.d.'s is used for estimating e.s.d.'s involving l.s. planes.
Refinement. Refinement of *F*^2^ against ALL reflections. The weighted *R*-factor *wR* and goodness of fit *S* are based on *F*^2^, conventional *R*-factors *R* are based on *F*, with *F* set to zero for negative *F*^2^. The threshold expression of *F*^2^ >σ2(*F*^2^) is used only for calculating *R*-factors(gt) *etc*. and is not relevant to the choice of reflections for refinement. *R*-factors based on *F*^2^ are statistically about twice as large as those based on *F*, and *R*- factors based on ALL data will be even larger.

### Fractional atomic coordinates and isotropic or equivalent isotropic displacement parameters (Å^2^)

**Table:**

	*x*	*y*	*z*	*U*_iso_*/*U*_eq_	
Br1	0.398775 (17)	0.50046 (2)	0.33147 (2)	0.03521 (8)	
O1	0.39056 (11)	−0.15520 (13)	0.34637 (12)	0.0279 (3)	
O2	0.29976 (11)	−0.10151 (12)	0.45837 (12)	0.0242 (3)	
C1	0.22157 (16)	−0.24807 (18)	0.55731 (16)	0.0229 (4)	
C2	0.11279 (15)	−0.16903 (17)	0.48370 (16)	0.0206 (4)	
C3	−0.00178 (16)	−0.18682 (18)	0.48681 (17)	0.0247 (4)	
C4	−0.04236 (18)	−0.3253 (2)	0.47273 (18)	0.0271 (4)	
C5	−0.06898 (17)	−0.3574 (2)	0.33847 (18)	0.0263 (4)	
C6	−0.04154 (16)	−0.23344 (18)	0.29110 (17)	0.0242 (4)	
C7	0.08449 (15)	−0.20150 (17)	0.34772 (16)	0.0202 (4)	
C8	0.17988 (15)	−0.29568 (18)	0.36060 (17)	0.0215 (4)	
C9	0.17465 (16)	−0.41722 (18)	0.42422 (18)	0.0245 (4)	
C10	0.19923 (16)	−0.38922 (18)	0.53953 (18)	0.0256 (4)	
C11	0.27888 (16)	−0.23479 (17)	0.47187 (17)	0.0224 (4)	
C12	−0.07986 (17)	−0.1352 (2)	0.35926 (19)	0.0283 (4)	
C13	0.35672 (14)	−0.07544 (18)	0.39376 (16)	0.0220 (4)	
C14	0.37096 (14)	0.06479 (18)	0.38565 (16)	0.0205 (4)	
C15	0.41471 (16)	0.1085 (2)	0.30735 (18)	0.0270 (4)	
C16	0.42547 (16)	0.2376 (2)	0.29232 (18)	0.0282 (4)	
C17	0.39139 (15)	0.32290 (19)	0.35609 (17)	0.0251 (4)	
C18	0.35028 (16)	0.28140 (19)	0.43685 (18)	0.0256 (4)	
C19	0.33979 (16)	0.15274 (19)	0.45069 (17)	0.0235 (4)	
H1	0.2663 (16)	−0.2218 (18)	0.6399 (18)	0.023 (5)*	
H2	0.1343 (15)	−0.0796 (19)	0.4998 (16)	0.017 (5)*	
H3	−0.0063 (16)	−0.143 (2)	0.5549 (18)	0.026 (5)*	
H4A	−0.1142 (19)	−0.326 (2)	0.4858 (19)	0.038 (6)*	
H4B	0.0117 (17)	−0.381 (2)	0.5314 (18)	0.025 (5)*	
H5A	−0.1481 (19)	−0.375 (2)	0.2916 (19)	0.032 (6)*	
H5B	−0.0256 (18)	−0.431 (2)	0.3317 (19)	0.033 (6)*	
H6	−0.0785 (18)	−0.225 (2)	0.203 (2)	0.034 (6)*	
H7	0.0949 (16)	−0.1252 (19)	0.3064 (17)	0.021 (5)*	
H8	0.1927 (17)	−0.3042 (19)	0.2898 (19)	0.030 (5)*	
H9	0.1568 (18)	−0.498 (2)	0.3870 (19)	0.032 (6)*	
H10	0.1999 (19)	−0.444 (2)	0.601 (2)	0.039 (6)*	
H11	0.3488 (18)	−0.279 (2)	0.4991 (18)	0.030 (6)*	
H12A	−0.1611 (19)	−0.143 (2)	0.3404 (19)	0.035 (6)*	
H12B	−0.0617 (18)	−0.050 (2)	0.3443 (18)	0.030 (5)*	
H15	0.4371 (18)	0.046 (2)	0.2652 (19)	0.031 (6)*	
H16	0.4559 (18)	0.268 (2)	0.2405 (19)	0.031 (6)*	
H18	0.3300 (17)	0.338 (2)	0.4785 (19)	0.029 (6)*	
H19	0.3133 (16)	0.1248 (19)	0.5017 (18)	0.022 (5)*	

### Atomic displacement parameters (Å^2^)

**Table:**

	*U*^11^	*U*^22^	*U*^33^	*U*^12^	*U*^13^	*U*^23^
Br1	0.03566 (13)	0.02801 (12)	0.04882 (14)	0.00147 (9)	0.02486 (10)	0.01104 (10)
O1	0.0274 (7)	0.0271 (8)	0.0316 (8)	−0.0003 (6)	0.0152 (6)	−0.0057 (6)
O2	0.0253 (7)	0.0204 (7)	0.0312 (7)	−0.0038 (5)	0.0163 (6)	−0.0024 (5)
C1	0.0270 (10)	0.0230 (10)	0.0163 (9)	−0.0028 (8)	0.0072 (8)	−0.0019 (7)
C2	0.0246 (9)	0.0169 (9)	0.0204 (9)	−0.0021 (7)	0.0101 (7)	−0.0015 (7)
C3	0.0284 (10)	0.0245 (10)	0.0245 (10)	−0.0017 (9)	0.0147 (8)	−0.0039 (8)
C4	0.0296 (10)	0.0272 (11)	0.0270 (10)	−0.0055 (9)	0.0146 (8)	0.0000 (8)
C5	0.0240 (10)	0.0263 (11)	0.0257 (10)	−0.0058 (9)	0.0081 (8)	−0.0034 (8)
C6	0.0244 (10)	0.0255 (10)	0.0192 (9)	−0.0008 (8)	0.0064 (8)	0.0031 (7)
C7	0.0243 (9)	0.0181 (9)	0.0188 (9)	−0.0002 (7)	0.0101 (7)	0.0029 (7)
C8	0.0261 (10)	0.0205 (9)	0.0202 (9)	−0.0001 (8)	0.0123 (8)	−0.0012 (7)
C9	0.0261 (10)	0.0176 (9)	0.0290 (10)	−0.0005 (8)	0.0114 (8)	−0.0024 (8)
C10	0.0272 (10)	0.0209 (10)	0.0263 (10)	0.0004 (8)	0.0096 (8)	0.0058 (8)
C11	0.0240 (10)	0.0169 (9)	0.0263 (10)	−0.0008 (8)	0.0111 (8)	−0.0006 (7)
C12	0.0248 (11)	0.0252 (11)	0.0342 (11)	0.0011 (8)	0.0124 (9)	0.0016 (9)
C13	0.0153 (9)	0.0279 (10)	0.0187 (9)	−0.0018 (8)	0.0036 (7)	−0.0011 (8)
C14	0.0138 (9)	0.0263 (10)	0.0183 (9)	−0.0006 (7)	0.0043 (7)	0.0006 (7)
C15	0.0227 (10)	0.0325 (12)	0.0291 (10)	0.0005 (8)	0.0145 (8)	−0.0027 (9)
C16	0.0248 (10)	0.0340 (12)	0.0308 (11)	−0.0016 (9)	0.0167 (9)	0.0047 (9)
C17	0.0190 (9)	0.0261 (10)	0.0277 (10)	0.0004 (8)	0.0080 (8)	0.0061 (8)
C18	0.0248 (10)	0.0270 (11)	0.0274 (10)	0.0015 (8)	0.0136 (8)	−0.0002 (8)
C19	0.0230 (10)	0.0275 (11)	0.0226 (9)	−0.0017 (8)	0.0124 (8)	0.0030 (8)

### Geometric parameters (Å, º)

**Table:**

Br1—C17	1.8986 (19)	C7—C8	1.558 (3)
O1—C13	1.211 (2)	C7—H7	0.989 (19)
O2—C13	1.338 (2)	C8—C9	1.512 (3)
O2—C11	1.450 (2)	C8—C11	1.553 (3)
C1—C10	1.509 (3)	C8—H8	0.96 (2)
C1—C11	1.542 (2)	C9—C10	1.331 (3)
C1—C2	1.562 (3)	C9—H9	0.95 (2)
C1—H1	0.96 (2)	C10—H10	0.94 (2)
C2—C3	1.547 (3)	C11—H11	0.96 (2)
C2—C7	1.570 (2)	C12—H12A	1.00 (2)
C2—H2	0.977 (19)	C12—H12B	0.96 (2)
C3—C4	1.534 (3)	C13—C14	1.494 (3)
C3—C12	1.541 (3)	C14—C19	1.393 (3)
C3—H3	0.98 (2)	C14—C15	1.395 (3)
C4—C5	1.554 (3)	C15—C16	1.384 (3)
C4—H4A	1.03 (2)	C15—H15	0.96 (2)
C4—H4B	0.96 (2)	C16—C17	1.386 (3)
C5—C6	1.532 (3)	C16—H16	0.94 (2)
C5—H5A	0.97 (2)	C17—C18	1.389 (3)
C5—H5B	0.98 (2)	C18—C19	1.377 (3)
C6—C7	1.539 (3)	C18—H18	0.90 (2)
C6—C12	1.545 (3)	C19—H19	0.89 (2)
C6—H6	0.97 (2)		
			
C13—O2—C11	116.73 (14)	C9—C8—C7	111.72 (15)
C10—C1—C11	96.63 (15)	C11—C8—C7	99.44 (14)
C10—C1—C2	111.48 (15)	C9—C8—H8	116.9 (12)
C11—C1—C2	99.63 (14)	C11—C8—H8	114.3 (12)
C10—C1—H1	115.2 (12)	C7—C8—H8	115.3 (12)
C11—C1—H1	115.1 (12)	C10—C9—C8	108.14 (17)
C2—C1—H1	116.1 (12)	C10—C9—H9	126.9 (13)
C3—C2—C1	126.18 (16)	C8—C9—H9	125.0 (13)
C3—C2—C7	102.57 (14)	C9—C10—C1	108.11 (17)
C1—C2—C7	102.99 (14)	C9—C10—H10	128.1 (14)
C3—C2—H2	108.3 (11)	C1—C10—H10	123.8 (14)
C1—C2—H2	106.3 (11)	O2—C11—C1	109.83 (14)
C7—C2—H2	109.6 (11)	O2—C11—C8	115.36 (15)
C4—C3—C12	100.00 (16)	C1—C11—C8	94.35 (14)
C4—C3—C2	114.30 (16)	O2—C11—H11	107.6 (13)
C12—C3—C2	99.46 (15)	C1—C11—H11	114.3 (12)
C4—C3—H3	113.4 (12)	C8—C11—H11	115.1 (13)
C12—C3—H3	115.3 (12)	C3—C12—C6	94.28 (15)
C2—C3—H3	113.0 (12)	C3—C12—H12A	112.4 (12)
C3—C4—C5	103.31 (15)	C6—C12—H12A	113.3 (12)
C3—C4—H4A	107.0 (12)	C3—C12—H12B	113.9 (13)
C5—C4—H4A	111.3 (12)	C6—C12—H12B	110.4 (13)
C3—C4—H4B	112.8 (12)	H12A—C12—H12B	111.5 (18)
C5—C4—H4B	113.7 (12)	O1—C13—O2	124.28 (17)
H4A—C4—H4B	108.5 (16)	O1—C13—C14	124.53 (17)
C6—C5—C4	103.38 (16)	O2—C13—C14	111.18 (16)
C6—C5—H5A	108.5 (13)	C19—C14—C15	119.22 (18)
C4—C5—H5A	110.6 (12)	C19—C14—C13	122.57 (17)
C6—C5—H5B	112.8 (13)	C15—C14—C13	118.19 (17)
C4—C5—H5B	112.8 (12)	C16—C15—C14	120.73 (19)
H5A—C5—H5B	108.7 (18)	C16—C15—H15	121.7 (13)
C5—C6—C7	113.99 (16)	C14—C15—H15	117.6 (13)
C5—C6—C12	100.17 (16)	C15—C16—C17	118.77 (19)
C7—C6—C12	99.27 (15)	C15—C16—H16	121.4 (13)
C5—C6—H6	114.0 (13)	C17—C16—H16	119.9 (13)
C7—C6—H6	113.5 (12)	C16—C17—C18	121.42 (19)
C12—C6—H6	114.2 (13)	C16—C17—Br1	119.62 (15)
C6—C7—C8	125.45 (16)	C18—C17—Br1	118.95 (15)
C6—C7—C2	103.35 (14)	C19—C18—C17	119.16 (19)
C8—C7—C2	102.95 (14)	C19—C18—H18	120.7 (13)
C6—C7—H7	108.4 (11)	C17—C18—H18	120.1 (13)
C8—C7—H7	106.1 (11)	C18—C19—C14	120.66 (18)
C2—C7—H7	110.0 (11)	C18—C19—H19	120.2 (13)
C9—C8—C11	96.33 (14)	C14—C19—H19	119.1 (13)
			
C10—C1—C2—C3	51.8 (2)	C13—O2—C11—C1	175.24 (15)
C11—C1—C2—C3	152.94 (17)	C13—O2—C11—C8	−79.7 (2)
C10—C1—C2—C7	−64.62 (18)	C10—C1—C11—O2	173.37 (14)
C11—C1—C2—C7	36.49 (16)	C2—C1—C11—O2	60.18 (17)
C1—C2—C3—C4	−48.0 (2)	C10—C1—C11—C8	54.41 (15)
C7—C2—C3—C4	68.68 (19)	C2—C1—C11—C8	−58.77 (15)
C1—C2—C3—C12	−153.53 (17)	C9—C8—C11—O2	−168.60 (15)
C7—C2—C3—C12	−36.89 (17)	C7—C8—C11—O2	−55.28 (18)
C12—C3—C4—C5	36.59 (19)	C9—C8—C11—C1	−54.24 (15)
C2—C3—C4—C5	−68.6 (2)	C7—C8—C11—C1	59.08 (15)
C3—C4—C5—C6	−0.5 (2)	C4—C3—C12—C6	−57.68 (17)
C4—C5—C6—C7	69.3 (2)	C2—C3—C12—C6	59.25 (16)
C4—C5—C6—C12	−35.73 (19)	C5—C6—C12—C3	57.45 (17)
C5—C6—C7—C8	48.1 (2)	C7—C6—C12—C3	−59.15 (16)
C12—C6—C7—C8	153.65 (17)	C11—O2—C13—O1	0.4 (2)
C5—C6—C7—C2	−68.78 (19)	C11—O2—C13—C14	179.24 (14)
C12—C6—C7—C2	36.79 (17)	O1—C13—C14—C19	−173.99 (17)
C3—C2—C7—C6	−0.02 (17)	O2—C13—C14—C19	7.2 (2)
C1—C2—C7—C6	132.22 (15)	O1—C13—C14—C15	7.6 (3)
C3—C2—C7—C8	−131.80 (15)	O2—C13—C14—C15	−171.21 (16)
C1—C2—C7—C8	0.43 (17)	C19—C14—C15—C16	−1.1 (3)
C6—C7—C8—C9	−53.2 (2)	C13—C14—C15—C16	177.33 (17)
C2—C7—C8—C9	63.86 (18)	C14—C15—C16—C17	−0.3 (3)
C6—C7—C8—C11	−153.94 (16)	C15—C16—C17—C18	2.0 (3)
C2—C7—C8—C11	−36.89 (16)	C15—C16—C17—Br1	−177.12 (15)
C11—C8—C9—C10	35.27 (19)	C16—C17—C18—C19	−2.2 (3)
C7—C8—C9—C10	−67.5 (2)	Br1—C17—C18—C19	176.96 (14)
C8—C9—C10—C1	0.1 (2)	C17—C18—C19—C14	0.6 (3)
C11—C1—C10—C9	−35.77 (19)	C15—C14—C19—C18	1.0 (3)
C2—C1—C10—C9	67.3 (2)	C13—C14—C19—C18	−177.42 (17)

### Possible structure/reactivity relationships (°, Å)

**Table:**

	1-OPBB	2-OPBB	3-OPBB	4-OPBB	5-OPBB
Solvolysis rate*^a^*	210	480	28	1.0	10^-11^
1:2 interplanar angle*^b^*	121.9 (2)	119.8 (6)	122.9 (3)	124.5 (1)	121.2 (1)
3:4 interplanar angle	132.0 (1)	132.4 (4)	128.1 (2)		
C11—O2 bond length*^b^*	1.450 (2)	1.460 (7)	1.437 (3)	1.445 (2)	1.447 (2)

Notes: (*a*) Rates determined in 80% dioxane-*d*_8_/20% D_2_O at 383 K, from NMR peak integrations. (*b*) C1/C7/C4 is plane 1 and C1/C2/C3/C4 is plane 2 for 4-OPBB and 5-OPBB. Bond length is C7—O2 for 4-OPBB and 5-OPBB.
